# Prevalence of obesity in a rural Asian Indian (Bangladeshi) population and its determinants

**DOI:** 10.1186/s12889-015-2193-4

**Published:** 2015-09-04

**Authors:** Tasnima Siddiquee, Bishwajit Bhowmik, Nayla Cristina Da Vale Moreira, Anindita Mujumder, Hajera Mahtab, A. K. Azad Khan, Akhtar Hussain

**Affiliations:** Department of International Health, Institute of Health and Society, Faculty of Medicine, University of Oslo, P.O. Box 1130, Blindern, N-0317 Oslo Norway; Department of Endocrinology, Bangladesh Institute of Research and Rehabilitation in Diabetes, Endocrine and Metabolic Disorders (BIRDEM), Dhaka, 1200 Bangladesh; Department of Pathology, Ibrahim Medical College, Diabetic Association of Bangladesh, Dhaka, 1200 Bangladesh

## Abstract

**Background:**

Obesity has reached epidemic proportions worldwide including Bangladesh. To assess the prevalence and associated factors of general and central obesity in a rural Bangladeshi population based on newly proposed cut off level for Asian population.

**Methods:**

2293 subjects aged ≥20 years from rural Bangladesh were randomly recruited to participate in a population-based, cross sectional survey, conducted in 2009. Both socio-demographic and anthropometric measurements were recorded. Age adjusted data for anthropometric indices were examined.

**Results:**

The age standardized prevalence of overweight (BMI 23-24.9 kg/m^2^) and obesity (BMI ≥25 kg/m^2^) were 17.7 (95 % confidence interval (CI): 16.1, 19.2 %) and 26.2 % (95 % CI: 24.4, 27.9 %), respectively. The age standardized prevalence of central obesity based on WC (M ≥90 & F ≥80 cm) and WHR (M ≥0.90 & F ≥0.80) were 39.8 % (95 % CI: 37.9, 41.7 %) and 71.6 % (95 % CI: 69.8, 73.4 %) respectively. The result shows that prevalence of central obesity was more in female than male. Study shows middle age, medium and high socioeconomic status (SES), low education levels, physical inactivity, high consumption of carbohydrate, protein and fat, were significant risk indicators for general and central obesity. Smoking was shown as protective factor for both general and central obesity.

**Conclusions:**

In rural Bangladeshi population, the prevalence of both general and central obesity was high among both sexes with the use of newly proposed cut off points for Asian population. Gender, diet, physical activity, education levels and SES were associated with the increase prevalence of obesity.

## Background

Obesity in a major public health concern of the twenty first century because of its alarming upward trend in both developed and developing countries [1, 2]. Obesity is already among the top 10 risks to human health worldwide [3]. According to the WHO report, one in three of the world's adult population is overweight and almost one in 10 is obese. Additionally there are over 20 million children under age five who are overweight [4]. Epidemiological survey used body mass index (BMI) as a measure of general obesity, and waist circumference (WC) and waist hip ratio (WHR) as measures of central/abdominal obesity. Both general and central obesity have been associated with a number of cardiometabolic abnormalities including prediabetes, type 2 diabetes (T2DM), hypertension (HTN), metabolic syndrome (MS) and cardiovascular diseases (CVDs) [5, 6]. Studies in Bangladeshi population have also found similar association at lower BMI, WC and WHR levels [7–9]. The consequence of obesity is not only limited to health consequences but also has an immense effect on individual and national healthcare budget.

Population-based data on the prevalence of obesity and associated factors in Bangladeshi adults have been limited until recently. In 2010, WHO estimated the prevalence of over-weight/obesity (BMI ≥25 kg/m^2^) aged over 15 was 8.4 % in Bangladesh [10]. In another study assessed the prevalence of overweight and obesity among women of reproductive age in South Asia between 1996-2006 also reported increase trends of prevalence. Overweight/obesity prevalence increased from 2.7 % to 8.9 % among Bangladeshi women in 10 years [11]. A recent urban study in Bangladesh has also found increased prevalence of overweight and obesity and it was much higher among those with higher socioeconomic status [12].

Bangladesh is an agro-based rural country where a vast majority (72 %) of the national population lives in rural areas [13], it is important to collect data on the prevalence of obesity and its associated factors. The purposes of the present study are to assess the prevalence of both general and central obesity and their associated factors in a rural Bangladeshi population.

We used the Asian-specific definition to define the prevalence of general and central obesity as it recently suggested by the International Association for the Study of Obesity and the International Obesity Task Force and International Diabetes Federation because of the observed differences in South Asian population [14–16].

## Methods

### Study design and participant selection

This study was part of a large longitudinal epidemiological study on diabetes and related cardiometabolic diseases in rural Bangladesh which has been described previously [17]. This population-based cross-sectional study was conducted in a rural community called Chandra, 40 km. north of Bangladesh’s capital, Dhaka during March to December 2009. A total of 10 villages were randomly selected from 25 villages with a population of approximately 20,000 aged ≥20 years. In order to determine the required sample size, the formula: *n* = Z^2^PQ/d^2^ was used, where, Z = 1.96, P for prevalence (DM & impaired glucose regulation) rural area from the previous study [18] i.e. 0.15; Q = 1-P i.e. 0.85 and d = allowable error of known prevalence i.e. 0.09 x 0.15. Ideally, it should be 0.05 x 0.15; but, to be on safe estimation with minimum sample size we allowed only 9 % (or .09) error of prevalence. Thus the calculated sample size was, *n* = 2687. Around 3000 individuals (both male and female) aged ≥20 years of those 10 villages were invited to participate in this study by following simple random procedure from the record of the voters lists number; among them 2376 (79.2 %) agreed individuals were investigated. Regarding non-response, survey timetable in early morning with 2-3 h study time and secondly, the improvement of healthcare facilities in Government Community Clinics may reduce the interest in obtaining a health assessment through the survey. This epidemiological survey was conducted in 20 selected spots of 10 villages. The inclusion criteria's were: both gender, aged ≥20 years and willing to participate. Exclusion criteria included pregnant women, and subjects with self-reported or medical history of myocardial infarction, renal disease, liver disease, tuberculosis, malignant disease and any severe infection at the time of screening.

### Data collection and measurements

Once the selection procedure was completed, participants were requested to visit a nearby field center. The socio-demographic data was collected by interviewing the participants using a predesigned pretested questionnaire. Survey procedures included completing a questionnaire on socio-demographic, physical activity, dietary and smoking information, anthropometric measurements, blood pressure measurement and laboratory investigations. Anthropometric measurements, such as height, weight and waist and hip circumferences, were taken with the participants wearing light clothes and without shoes. The weight was taken to the nearest 0.1 kg by modern electronic digital LCD weighing machines (Best Deluxe Model; Bathroom, Dhaka, Bangladesh) placed on a flat surface. The scales were calibrated everyday against a standard (20 kg). Height was taken while the participants stood in erect posture, touching the occiput, back, hip and heels on a straight measuring wall, while the participants looked straight ahead. BMI was calculated as the weight (kg) divided by square of the height (m^2^). Waist circumference was measured by placing a tape horizontally midway between the lower margin of the last palpable rib and iliac crest on the mid-axillary line. Hip circumference was measured at a level parallel to the floor, at the largest circumference of the buttocks. WHR was then calculated from waist and hip circumference (cm). Food consumption and portion sizes were calculated with the 24-h recall method. Participants were asked to report on all foods and drinks consumed in the previous 24 h (the previous day), in direct chronological order from the first foods in the morning to the last foods before going to bed. A range of local household utensils: glasses, spoons, cups and plates were used for estimating the amount of foods and beverages actually consumed by the respondents. The use of these local utensils acted as visual aids to increase the accuracy of portion size estimations. In some instances the respondent was asked to supply her own utensils for the recall. To obtain the weight/g equivalents of foods, the Bangladesh Food Photo Manual was used, which converts food items of different sizes and composition to gram equivalents. In addition, the respondents were asked to provide information on the following: type of fat/oil used for cooking, preferred cooking method, meal consumption patterns, number of family members use the same kitchen. National Cholesterol Education Program guideline was followed for daily intake of carbohydrate (>55 %), protein (15 %) and fat (>30 %) [19].

### Definitions

Cut-off values for overweight and general obesity for both sexes were BMI 23-24.9 kg/m^2^ and BMI of ≥25 kg/m^2^ respectively; cut-off values for central obesity including waist circumference for male and female were ≥90 and ≥80 cm, WHR for male and female were ≥0.90 and ≥0.80 [14, 15]. Based on the monthly expenditure socio-economic condition was classified as low (<6000 Bangladeshi Taka [BDT, 1 USD = 84 BDT]), medium (6000-11000 BDT) and high (>11000 BDT). Education level graded as illiterate: unable to write and read; having primary and secondary education and college and above. Physical activity was graded on the ordinal scale of 1-3, corresponding to light, moderate and heavy, according to the activity level based on their occupation. For the purpose of data analysis, these results were transformed into a binary variable - inactive (grade1) and active (grade 2 and 3). Smoking habit was classified as either current or non/ex-smoker.

### Ethics statement

The study was carried out in accordance with the declaration of Helsinki as revised in 2000 and all procedures involving for this study were approved by Regional Ethics Committee (REK) of Norway and the Ethics Review Committee (ERC) of Diabetic Association of Bangladesh for Medical Research. Since approximately 52.1 % of the adult population is illiterate [13], an informed verbal consent was received from all subjects or guardians of the subjects prior to inclusion in the study to avoid selection bias. The participants were also verbally informed of their right to withdraw from the study at any stage, or to restrict their data from the analysis. After the verbal information, a printed copy of their rights was given. Both the Ethics Review Committees were satisfied with the voluntary participation, maintenance of the rights of the participating subjects and confidential handling of personal information by the study team and has approved the consent procedure.

### Statistical analysis

The present analysis is based on 2,293 participants (842 male and 1,451 female) for whom all the variables were available. Both STATA 11 for Windows (STATA Co., College Station, TX, USA) and PASW statistics version 18 for Windows (SPSS Inc., Chicago, IL, USA) were used as needed. Means and Percentages with 95 % confidence intervals adjusted for age were given for normally distributed continuous variables and categorical variables as needed. Age specific and age standardized prevalence by direct standardization method were estimated on the basis of 2001 census data before performing statistical tests [20]. Differences between the groups of means and proportions adjusted for age were tested by analysis of covariance (ANCOVA) and logistic regression. Chi-squared test was used to test the trend. In the analysis, general (defined by BMI) and central obesity (defined by WC) were considered as the dependent variable. Socio- demographic and dietary factors were included in the regression analysis to identify variables independently associated with overweight and obesity. In the analysis, only those variables which were identified to be independently associated in univariate analyses were included in multinomial models. Statistical inference is based on 95 % confidence intervals (CIs) and the significance level was set at 0.05.

## Results

Demographic and socio-economic characteristics of the study populations was illustrated in Table 1. Among the total number of participants, 36.7 % (*n* = 842) were male and 63.3 % (*n* = 1451) were female participants. Females were younger as opposed to male subjects. Among the participants, 23.9, 30.6, 24.5 and 21 % were aged between 20 to 30 years, 31 to 40 years, 41 to 50 years and ≥51 years respectively. A major portion of the participants were Illiterate (45.2 %), and housewives (58.7 %), and 42.2 % and 41.6 % were from middle and low income groups respectively. 15.9 % and 15.1 % participants were cigarette smokers and physically inactive respectively. Mean calorie consumption was 1600 Kcal/day. 90.6, 32.9 and 8.5 % participants consumed recommended level of carbohydrate (>55 %), protein (≥15 %) and fat (>30 %) respectively.

**Table 1:** Demographic and socio-economic characteristics of the study subjects (*n* = 2293)

Variables		Number	
Age	Total Participants	2293	41.8 (41.2, 42.4)
	Male	842	44.3 (43.3, 45.2)
	Female	1451	40.4 (39.7, 41.1)
Age group (year), %	20-30	548	23.9
	31-40	702	30.6
	41-50	562	24.5
	≥51	481	21.0
Education, %	Illiterate	1037	45.2
	Primary (0-5 class)	419	18.3
	Secondary (6-10 class)	627	27.3
	College (>10 class) & above	210	9.2
Occupation, %	Farmer	443	19.3
	Business	199	8.7
	Skill labour	112	4.8
	Manual labour	194	8.5
	Housewives	1345	58.7
Socioeconomic status, %	Low income (<6000 BDT)	953	41.6
	Middle income (6000-11000 BDT)	967	42.2
	High income (>11000 BDT)	373	16.3
Smoking habit, %	Cigarette smoking	365	15.9
Physical activity, %	Active	1946	84.9
	Inactive	347	15.1
Calorie consumption	Total calorie intake (kcal/day)	2293	1600 (1587-1613)
	Carbohydrate (>55 %)	2077	90.6
	Protein (≥15 %)	754	32.9
	Fat (>30 %)	196	8.5

Data expressed as % or mean ± 95 % CI. CI, confidence interval

Age specific and age standardized prevalence of BMI levels were shown in Table 2. The age standardized prevalence of underweight, normal weight, overweight and obese were 14.3 %, 41.9 %, 17.7 %, and 26.2 %, respectively. No significant sex difference was observed for the prevalence of BMI levels. With increasing age in female, underweight group showed a significant increasing trend of low BMI.

**Table 2:** Age specific and age standardized prevalence of different Body Mass Index (BMI) levels (*n* =2293)

Variables	Age specific (year) prevalence, %				Age Standardized prevalence, % (95 % CI)
	20-30 (*n* = 548)	31-40 (*n* = 702)	41-50 (*n* = 562)	≥51 (*n* = 481)	20-80 years
Underweight (BMI <18.5 Kg/m^2^)					
Total (*n* = 328)	11.7	10.3	16.2	21.0	14.3 (12.9, 15.7)
Male	14.6	9.1	14.3	18.6	13.6 (11.3, 16.0)
Female	10.4	10.9	17.5	23.1^a^	14.9 (12.1, 16.8)
Normal weight (BMI 18.5- < 23 Kg/m^2^)					
Total (*n* = 960)	46.2	39.2	41.1	41.8	41.9 (39.9, 43.9)
Male	44.2	41.4	41.1	42.9	42.3 (38.9, 45.7)
Female	47.0	38.9	41.1	40.8	41.5 (38.9, 44.1)
Overweight (BMI 23- < 25 Kg/m^2^)					
Total (*n* = 405)	18.6	17.1	17.6	17.5	17.7 (16.1, 19.2)
Male	18.8	19.8	17.9	19.9	19.1 (16.5, 21.8)
Female	18.5	15.7	17.5	15.4	16.8 (14.9, 18.7)
Obese (BMI ≥25 Kg/m^2^)					
Total (*n* = 600)	23.7	33.5	25.1	19.8	26.2 (24.4, 27.9)
Male	22.4	29.7	26.8	18.6	25.1 (22.1, 28.0)
Female	24.0	35.3	23.9	20.8	26.8 (24.5, 29.0)

Values are presented as % (number) or (95 % confidence interval) as indicated. Age adjustment was based on 2001 census of Bangladesh

^a^χ^2^ trend = *P* < 0.05 for different age group. CI, confidence interval; BMI, body mass index

Age specific and age adjusted mean of body mass index (BMI) by different levels were shown in Table 3. The age adjusted mean of underweight, normal weight, overweight, obese and overall were 17.1, 20.8, 23.9, 27.7 and 22.4 kg/m^2^ respectively. Significant difference between the two sexes was noticed in the age adjusted mean of obese group (27.3 kg/m^2^ in male and 27.9 kg/m^2^ in female; *P* < 0.05).

**Table 3:** Age specific and Age adjusted mean of Body Mass Index (BMI) by different levels (*n* = 2293)

	Age specific (year) means (95 % CI)				Age adjusted mean (95 % CI)
	20-30 (*n* = 548)	31-40 (*n* = 702)	41-50 (*n* = 562)	≥51 (*n* = 481)	
Underweight (*N* =328)					
Total	17.1 (16.8, 17.3)	17.5 (17.3, 17.7)	17.1 (16.9, 17.4)	16.7 (16.5, 16.9)	17.1 (16.9, 17.2)
Male	17.0 (16.5, 17.5)	17.6 (17.2, 18.1)	17.2 (16.7, 17.7)	16.8 (16.4, 17.2)	17.1 (16.9, 17.3)
Female	17.1 (16.8, 17.4)	17.5 (17.2, 17.7)	17.1 (16.8, 17.4)	16.7 (16.4, 17.2)	17.1 (16.9, 17.2)
Normal weight (*N* = 960)					
Total	20.8 (20.6, 20.9)	20.8 (20.6, 20.9)	20.9 (20.7, 21.0)	20.8 (20.6, 23.0)	20.8 (20.7, 20.9)
Male	20.7 (20.4, 21.0)	20.8 (20.6, 21.1)	20.8 (20.5, 21.1)	20.8 (20.5, 21.0)	20.8 (20.7, 20.9)
Female	20.8 (20.6, 21.0)	20.8 (20.6, 20.9)	20.9 (20.7, 21.1)	20.8 (20.6, 21.1)	20.8 (20.7, 20.9)
Overweight (*N* = 405)					
Total	23.9 (23.8, 24.1)	23.9 (23.8, 24.1)	23.9 (23.8, 24.0)	23.9 (23.8, 24.0)	23.9 (23.8, 24.0)
Male	23.9 (23.7, 24.1)	23.9 (23.8, 24.2)	23.9 (23.8, 24.0)	23.9 (23.7, 24.0)	23.9 (23.8, 24.0)
Female	24.0 (23.8, 24.1)	24.0 (23.8, 24.1)	23.9 (23.8, 24.1)	23.9 (23.8, 24.0)	23.9 (23.8, 24.0)
Obese (*N* = 600)					
Total	27.6 (27.2, 28.0)	27.9 (27.6, 28.2)	27.6 (27.2, 28.0)	27.3 (26.8, 27.7)	27.7 (27.5, 27.9)
Male	27.3 (26.6, 27.9)	27.3 (26.9, 27.7)	27.4 (26.7, 28.1)	27.1 (26.3, 27.8)	27.3 (27.0, 27.6)
Female	27.3 (27.0, 28.3)	28.2 (27.8, 28.6)	27.7 (27.2, 28.2)	27.4 (26.9, 28.0)	27.9 (27.7, 28.1)^b^
Overall (*N* = 2293)					
Total	22.6 (22.2, 22.9)	23.4 (23.1, 23.7)	22.5 (22.2, 22.8)	21.8 (21.4, 22.1)	22.4 (22.3, 22.5)
Male	22.3 (21.7, 22.8)	23.1 (22.6, 23.5)	22.6 (22.1, 23.1)	21.8 (21.3, 22.3)	22.3 (22.2, 22.4)
Female	22.7 (22.3, 23.1)	23.5 (23.2, 23.9)	22.4 (21.9, 22.8)	21.7 (21.2, 22.2)	22.4 (22.3, 22.5)

Values are presented as mean (95 % confidence interval) as indicated

^b^ *P* < 0.05 between male and female in same group CI, confidence interval; BMI, body mass index

Age specific and age standardized prevalence of central obesity based on Waist circumference (WC) and Waist hip ratio (WHR) were shown in Table 4. The age standardized prevalence of central obesity following WC and WHR were 39.8 % and 71.6 % respectively. Central obesity by WC and WHR were more in female than male. Age specific prevalence of central obesity, for both WC and WHR showed significant sex differences. The prevalence of central obesity by WHR demonstrated that a significant increasing trend of central obesity among female subjects with increasing age.

**Table 4:** Age specific and age standardized prevalence of central obesity based on Waist circumference (WC) and Waist hip ratio (WHR)

Variables	Age specific (year) prevalence, %				Age Standardized prevalence, % (95 % CI)
	20-30 (*n* = 548)	31-40 (*n* = 702)	41-50 (*n* = 562)	≥51 (*n* = 481)	20-80 years
Central Obesity by WC (cm)					
Total (M ≥90 & F ≥80)	34.9	46.3	41.6	33.9	39.8 (37.9, 41.7)
Male (≥90 cm)	16.4	28.9	29.9	19.9	24.3 (21.4, 27.1)
Female (≥80 cm)	42.8	54.9	49.4	45.8	48.7 (46.2, 51.3)^b^
Central Obesity by WHR					
Total (M ≥0.90 & F ≥0.80)	66.6	73.1	73.8	72.6^a^	71.6 (69.8, 73.4)
Male (≥0.90)	43.6	64.7	63.4	60.6^a^	58.4 (55.2, 61.8)
Female (≥0.80)	76.5	77.2	80.8	82.7^a^	79.1 (76.9, 81.1)^b^

Values are presented as % (number) or (95 % confidence interval) as indicated. Age adjustment was based on 2001 census of Bangladesh

CI, confidence interval

^a^χ^2^ trend = *P* < 0.05 Central Obesity based on WC and WHR, ^b^ *P* < 0.05 between male and female same group

Age specific and age adjusted mean of waist circumference (WC) and waist hip ratio (WHR), by sex was shown in Table 5. The age adjusted mean of central obesity following WC and WHR were 80.5 cm and 0.88 respectively. Significant difference by gender was noted in the age adjusted mean of central obesity by WC (81.8 cm in male and 79.7 cm in female; *P* < 0.05) and WHR (0.91 in male and 0.86 in female; *P* < 0.05). This was also observed in age specific mean of central obesity, both WC and WHR groups including the mean of central obesity following WC and WHR among age groups.

**Table 5:** Age specific and age adjusted mean of Waist circumference (WC) and Waist hip ratio (WHR), by sex

Variables	Age specific (year) means, 95 % CI				Age adjusted
	20-30 (*n* = 548)	31-40 (*n* = 702)	41-50 (*n* = 562)	≥51 (*n* = 481)	Mean (95 % CI)
Central Obesity by WC (cm)					
Total (M ≥90 & F ≥80)	79.2 (78.4, 80.1)	81.8 (81.6, 82.6)	81.0 (80.1, 81.8)	79.5 (78.4, 80.4)^a^	80.5 (80.1, 80.9)
Male (≥90 cm)	80.0 (78.4, 81.5)	83.1 (81.9, 84.3)	82.8 (81.4, 84.1)	80.8 (79.4, 82.2)	81.8 (81.1, 82.5)
Female (≥80 cm)	78.9 (77.9, 79.9)	81.2 (80.2, 82.2)	79.8 (78.7, 80.9)	78.2 (76.8, 79.7)^a^	79.7 (79.2, 80.3)^b^
Central Obesity by WHR					
Total (M ≥0.90 & F ≥0.80)	0.86 (0.85, 0.87)	0.88 (0.86, 0.89)	0.89 (0.88, 0.90)	0.89 (0.88, 0.90)^a^	0.88 (0.87, 0.89)
Male (≥0.90)	0.89 (0.87, 0.90)	0.91 (0.90, 0.92)	0.92 (0.91, 0.93)	0.91 (0.90, 0.92)	0.91 (0.90, 0.93)
Female (≥0.80)	0.85 (0.84, 0.86)	0.86 (0.85, 0.87)	0.87 (0.86, 0.88)	0.87 (0.86, 0.88)^a^	0.86 (0.85, 0.87)^b^

Values are presented as mean (95 % confidence interval). Age adjustment was based on 2001 census of Bangladesh

CI, confidence interval. ^a^ *P* < 0.05 between age group: ^b^ *P* < 0.05 between male and female in same group

Table 6 showed middle age, medium and high SES, irrespective of education levels, physical inactivity, high consumption of carbohydrate, protein and fat, were some significant risk indicator for general and central obesity in both unadjusted and adjusted models. In addition, females had significant higher risk for central obesity. Smoking was found to have a protective effect for both general and central obesity.

**Table 6:** Association between general (BMI ≥25 Kg/m2) and central obesity (WC: M ≥90 & F ≥80) and socio-demographic and dietary factors in the surveyed population aged ≥20 years

	General obesity			Central obesity		
Variables	Unadjusted OR (95 % CI)	Adjusted OR (95 % CI)	*P* value	Unadjusted OR (95 % CI)	Adjusted OR (95 % CI)	*P* value
Age (years)						
20-30	Ref	Ref		Ref	Ref	
31-40	1.62 (1.26, 2.08)	1.63 (1.25, 2.13)	<0.001	1.61 (1.28, 2.02)	1.69 (1.32, 2.16)	<0.001
41-50	1.08 (0.82, 1.42)	1.22 (0.84, 1.49)	0.431	1.33 (1.05, 1.70)	1.56 (1.20, 2.03)	0.001
≥51	0.79 (0.59, 1.07)	0.75 (0.54, 1.03)	0.075	0.96, (0.74, 1.24)	1.05 (0.79, 1.38)	0.741
Sex						
Male	Ref	Ref		Ref	Ref	
Female	1.13 (0.93, 1.38)	1.05 (0.81, 1.35)	0.739	2.96 (2.45, 3.57)	3.70 (2.86, 4.79)	<0.001
Education						
Higher	Ref	Ref		Ref	Ref	
Secondary	2.85 (1.85, 4.39)	2.34 (1.49, 3.67)	<0.001	3.32 (2.32, 4.74)	2.13 (1.47, 3.91)	<0.001
Primary	2.81 (1.78, 4.47)	2.84 (1.79, 4.51)	<0.001	2.65 (1.79, 3.90)	2.65 (1.79, 3.91)	<0.001
Illiterate	2.33 (1.49, 3.65)	2.87 (1.86, 4.42)	<0.001	2.12 (1.47, 3.09)	3.32 (2.33, 4.74)	<0.001
SES						
Low	Ref	Ref		Ref	Ref	
Medium	1.68 (1.35, 2.08)	1.69 (1.35, 2.12)	<0.001	1.41 (1.17, 169)	1.59 (1.30, 1.94)	<0.001
High	3.62 (2.78, 4.70)	3.42 (2.60, 4.50)	<0.001	2.52 (1.98, 3.22)	2.91 (2.24, 3.81)	<0.001
Smoking						
No	Ref	Ref		Ref	Ref	
Yes	0.51 (0.38, 0.68)	0.48 (0.34, 0.68)	<0.001	0.75 (0.57, 0.96)	0.70 (0.51, 0.97)	0.031
Physical activity						
active	Ref	Ref		Ref	Ref	
inactive	1.42 (1.10, 1.82)	1.58 (1.17, 2.14)	0.003	1.80 (1.42, 2.48)	1.78 (1.32, 2.39)	<0.001
CHO intake (%)						
≤55 %	Ref	Ref		Ref	Ref	
>55 %	2.72 (1.99, 3.93)	2.78 (2.00, 4.00)	<0.001	2.97 (2.76, 3.72)	2.87 (2.04, 4.05)	<0.001
Protein intake (%)						
<15 %	Ref	Ref		Ref	Ref	
≥15 %	1.45 (1.11, 1.76)	1.25 (1.01, 1.55)	0.039	1.52 (1.25, 1.63)	1.26 (1.02, 1.56)	0.036
Fat intake (%)						
<30 %	Ref	Ref		Ref	Ref	
≥30 %	2.20 (1.35, 2.79)	1.78 (1.19, 2.65)	0.005	2.18 (1.85, 2.67)	1.78 (1.19, 2.67)	0.005

Adjusted by age, sec, educational status, socioeconomic status (SES), smoking, physical activity, depression, carbohydrate, protein and fat intake. BMI, body mass index; WC, waist circumference, SES, socio-economic status, CHO, carbohydrate

## Discussion

This current study was undertaken to explore the prevalence of overweight, obesity and central obesity (abdominal obesity) and their associated socio-demographic and lifestyle determinants in a rural Bangladeshi population aged ≥ 20 years and older.

For this current study, we have used newly proposed cut-off levels for Asian population for defining general and central obesity. Evidence shows that Asian Indians including Indian [21], Pakistani [22], Sri Lankan [23] and Bangladeshi [7–9] generally have a lower BMI than many other ethnic population, but the association between BMI and cardiometabolic risk factors is as strong as in any other population. The risk of diabetes and other cardiometabolic factors were significant for Bangladeshi populations with a BMI of >21 kg/m^2^ [7–9] and this has been confirmed by studies in other Asian populations [21–23]. WHO also recommend, a BMI of 18.5–22 kg/m^2^ is considered healthy for Asian populations [24]. Similarly, lower central obesity cut- off levels have also been recommended for the South Asians [16, 21].

The age standardized prevalence of overweight (BMI 23-24.9 kg/m^2^) and obesity (BMI ≥25 kg/m^2^) in current study were 17.7 and 26.2 % respectively. The prevalence of obesity documented in this study was comparatively higher than previous studies in Bangladesh conducted in different time points using different anthropometric cut-off levels [11, 25]. The rate was also found higher than rural areas of China, Greece and north India [26–28]; however, they used WHO cut-off levels for western population. In this study, age standardized prevalence of central obesity based on WC (M ≥90 & F ≥80 cm) and WHR (M ≥0.90 & F ≥0.80) were 39.8 % and 71.6 % respectively. The rate of central obesity was higher than general obesity in our study which indicate a significant portion of the population may not classified as obese on BMI levels. Hence, it has been suggested that a single BMI cut-off level for both male and female might not be adequate to define general obesity. Gender, age and ethnic specific BMI levels for defining general obesity might be preferable.

Study demonstrated that high prevalence of obesity was positively associated with female sex, middle age, higher educational and economic status, physical inactivity and some dietary habits in South Asian region [29]. For instance, similar findings were observe in our study except education level. In our study, the prevalence of obesity is slightly higher in female than male, while overweight is more prevalent in male. Our results are in agreements with the study conducted in Pakistan [30], South India [31] and Ghana [32] where prevalence of obesity was found to be higher among female than male. However, in Japan, male were more obese than female [33]. In the present analysis, rate of central obesity was also higher in female participants which is consistence with the study conducted in south India [31]. Increased parity, menopause, high rate of oral contraceptive pill intake and low level of cigarette smoking could be the possible contributors of high level of central obesity in females. Study findings indicate that our study population are at risk for cardiometabolic diseases. Findings emphasis the need for effective intervention with community based approaches to prevent and treat obesity.

We have observed that the highest prevalence of obesity in South Asians is observed in the middle aged (30-50 years) group, whereas in the western countries prevalence tends to increase progressively with age [34]. The highest prevalence was reached in the middle-aged (30-40 years) group in our study. In this study, we have found that people with lower levels of education and higher socioeconomic status had the higher rate of obesity. Similar finding was supported by other studies conducted in Australia, Greece, and Canada [35, 36, 28], although the cause in our study is not clear. Further long term well design study are needed to know the exact cause of this contrasting finding between education and SES. Possible hypothesis could be, majority of participants are farmers, businessmen, manual labors and housewives, and for these formal education are not mandatory. Due to continued economic growth and huge development in agricultural sectors in last few years now they have a good income. They can afford foods but due to lack of proper education they are not aware of healthy diet. They mainly eat rice, hydrogenated oil and sugar. All of these are known to increase the risk of weight gain. Evidence shows the global epidemic of obesity has resulted mainly from societal factors that promote increased availability of high-fat energy-dense foods, excess carbohydrate based diet, and physical inactivity. Physical inactivity, high intake of carbohydrate, protein and fat, were significant risk indicators for general and central obesity in our study.

Smoking was protective factor for general and central obesity in our study which is consistence with the studies were conducted in Australia, Portugal, Spain and Switzerland [35, 37–39]. Possible biological mechanism may explain the inverse association between smoking and obesity. Study has shown that smoking increase resting metabolic rate and thermogenesis, but also reduce energy intake and since it provides the smoker with a diminished sense of taste and smell, which makes food less attractive and therefore less is consumed which ultimately cause less weight gain [29].

The strength of the study that it was a large scale population based study where response rate was 79.0 %. Bias was taken care of by random sampling. Anthropometric measurements were done by the highly trained interviewers. The present study had several limitations. The major limitation was the cross-sectional design, which cannot establish causal relations. Therefore, we cannot say the identified risk factors are causally associated with both general and central obesity. Subject exclusion based on self-reported personal medical history was another limitation of the present study. The study was conducted in a rural area of Bangladesh adjacent to capital Dhaka city. Hence, the result may be interpreted with caution.

## Conclusion

It is apparent that obesity is increasing even in rural adult population. In rural Bangladeshi population, the rate of general and central obesity was high among both sexes with the use of newly proposed cut off points for Asian population. Central obesity was more in females than males. Gender, diet, physical activity, education level, SES, and smoking were associated with the prevalence of obesity. Along with socio-economic determinants, our rural population are less likely to receive counseling regarding healthy diet and exercise habits may place them in a more vulnerable position with respect to developing obesity over time. Nationally representative and longitudinal follow-up studies including all the possible influences are needed to confirm the risk indicators for obesity found in this study. The study indicates that intervention program is needed to identify practical, effective and acceptable methods for prevention of obesity. Finally, Government policy makers and other health related stakeholders should consider obesity as a growing public issue and therefore, an urgent need for national program to reduce obesity and associated comorbidity and mortality.
